# Strategies for Stabilization of Zn Anodes for Aqueous Zn-Based Batteries: A Mini Review

**DOI:** 10.3389/fchem.2021.822624

**Published:** 2022-02-09

**Authors:** Funian Mo, Ning He, Lina Chen, Mengrui Li, Suzhu Yu, Jiaolong Zhang, Wenhui Wang, Jun Wei

**Affiliations:** ^1^ Shenzhen Key Laboratory of Flexible Printed Electronics Technology Center, Harbin Institute of Technology, Shenzhen, China; ^2^ School of Materials Science and Engineering, Harbin Institute of Technology, Shenzhen, China; ^3^ School of Materials Science and Engineering, Dongguan University of Technology, Dongguan, China; ^4^ Department of Civil and Environmental Engineering, Harbin Institute of Technology, Shenzhen, China

**Keywords:** rechargeable Zn-based batteries, Zn anode, stabilization, dendrites, hydrogen evolution

## Abstract

In recent years, thanks to the investigation of the in-depth mechanism, novel cathode material exploitation, and electrolyte optimization, the electrochemical performance of rechargeable Zn-based batteries (RZBs) has been significantly improved. Nevertheless, there are still some persistent challenges locating the instability of the Zn anodes that hinder the commercialization and industrialization of RZBs, especially the obstinate dendrites and hydrogen evolution reaction (HER) on Zn anodes, which will dramatically compromise the cycle stability and Coulombic efficiency. Therefore, various strategies with fundamental design principles focusing on the suppression of dendrite and the HER have been carefully summarized and categorized in this review, which are critically dissected according to the intrinsic mechanisms. Finally, pertinent insights into the challenges and perspectives on the future development of Zn anodes are also emphasized, expecting to supply potential research directions to promote the practical applications of RZBs.

## Introduction

Rechargeable Zn-based batteries (RZBs) that possess synergistic advantages of intrinsic safety, environmental benignity, and satisfactory energy density have raised particular attention and been considered as a promising candidate for next-generation energy storage. Typically, RZBs consist of a metallic Zn anode, electrolyte, and an (in)organic cathode. Tremendous progress in recent years has been made for RZBs, which mainly focused on developing high-performance cathode materials (e.g., manganese-based oxides (Pan et al., 2016), vanadium-based oxides (Liu et al., 2020), Prussian blue analog-based compounds (Hurlbutt et al., 2018), and organic materials (Wan et al., 2018)) based on diverse energy storage mechanisms including chemical conversion, Zn^2+^ intercalation, co-insertion, and hybrid chemistry mechanism (Li et al., 2019). Additionally, electrolytes also play a key role in rechargeable Zn-based batteries, which can be divided into aqueous and non-aqueous electrolytes (e.g., organic electrolytes and ion liquid). The recent mainstream is the aqueous electrolytes, which are generally divided into neutral/mildly acid or alkaline based on pH. Through a series of systematic research for cathode materials exploration and electrolyte optimization, the electrochemical performances of RZBs have been significantly enhanced.

The Zn anode features the advantages of resource availability, low electrochemical potential (−0.76 V), and high theoretical capacity (820 mAh g^−1^), and its reaction can be generalized to be a zinc-plating/striping process (Chao et al., 2019; Jia et al., 2020). Nevertheless, the instability of the Zn anode and insufficient understanding of relative mechanisms have impeded RZBs from being deployed in their proposed commercialization and large-scale production. The main issues, from which the Zn anodes are suffering, can be categorized into two parts: dendrites and hydrogen evolution reaction (HER)/corrosion. Similar to many other metal anodes, the formation of Zn dendrites during the cyclic plating/stripping process is an intractable problem that results in capacity fading and eventual battery failure (Zhao J. et al., 2019; Yang Q. et al., 2020). On the other side, the HER and corrosion in aqueous electrolytes are also persistent challenges, which normally leads to the increased impedance, capacity deterioration, and electrolyte leakage (Ma et al., 2020). Worse of all, these issues are mutually reinforced that the byproducts originated from the HER and corrosion will be detrimental to the uniform ion transmission, thus inducing more dendrites. Meanwhile, the accumulated dendrites will increase specific areas on the Zn anode surface, exacerbating the HER and corrosion (Li et al., 2020a; Yi et al., 2021). If appropriate coping strategies can be developed to alleviate or even eliminate these problems, it will greatly enhance the battery performance of RZBs.

In this article, we first briefly summarize the fundamental mechanisms and effects of Zn dendrites and the HER in aqueous electrolytes. Second, with the aim of an integrated improvement, the corresponding stabilization strategies to suppress Zn dendrites, the HER, and corrosion are comprehensively summarized and discussed from perspectives of thermodynamics and kinetics. Finally, challenges and constructive perspectives of Zn anode optimization for the commercialization and industrialization of RZBs are proposed, with a hope to promote their practical applications.

## Fundamentals of Dendrite Formation and the Hydrogen Evolution Reaction

So far, Zn metal has been widely applied as anode materials for RZBs due to its intrinsic eco-friendliness and electrochemical stability. Many research studies based on experiments and simulations have been performed on the Zn anodes to achieve a systematic understanding of the electrochemical properties and associated influences on RZBs. However, it still suffers from some detrimental issues which are plaguing the scale-up implementation of RZBs. During the Zn plating/stripping process, the Zn dendrites would form on the surface of the Zn anode and increase its surface area. Additionally, HER/corrosion reactions would also occur on the metal Zn surface, causing a continuous consumption of active Zn and the increase of OH^−^ concentration. The evolved H_2_ on the anode surface would hinder Zn nucleation, leading to the increased overpotential and an uneven deposition. Meanwhile, the insoluble byproducts would form and adhere to the anode surface, resulting in the surface passivation of fresh Zn. This process would adversely affect the interface impedance between the electrolyte and the Zn anode, resulting in the increased electrode polarization. These irreversible side reactions of the HER, corrosion, and surface passivation, as well as Zn dendrite formation, generate a vicious circle and seriously affect the stability of the Zn anode (Tang et al., 2019). In this review, the persistent challenges of dendrite formation and the HER are comprehensively discussed.

### Fundamentals of Dendrite Formation

Under the electric field, the plating/stripping process of Zn ions is mainly commanded by the microenvironments of the anode and electrolyte including the surface polarization and 2D diffusion, as well as the mass transfer near the interface, resulting in the existence of original protrusions on the anode surface (Trócoli and La Mantia, 2015). These protrusions can be subsequently consolidated by the “tip effect” and grow perpendicularly to the surface to eventually formed dendrites (Zhang et al., 2020). Concentration polarization, which is referred to the electrochemical potential shift of the cell by comparing with its equilibrium value originated from the ion concentration difference between the electrolyte and the electrode surface, will further exacerbate dendrite issues at high current density (Yang et al., 2019). The growth of the Zn dendrite will ultimately make it pierce through the separator and cause short circuit.

### Fundamentals of the Hydrogen Evolution Reaction

The hydrogen evolution during Zn plating/stripping is originated from both H_2_O hydrolysis and zinc corrosion, which also normally occurs on the Zn anode surface. The fundamental reason of the hydrogen evolution is the thermodynamic instability of the Zn anode while working in an aqueous solution (Amiinu et al., 2017). In the mildly acidic/neutral electrolytes, the fundamental reaction of Zn anodes can be simply explained as the equation Zn=Zn^2+^+2e^−^. The reaction chemistry of the interfacial parasitic HER of Zn anodes can be depicted as 2H_2_O+2e^−^→H_2_↑+2OH^−^, and the formation of insoluble byproducts can be summarized as Zn^2+^+2e^−^→Zn(OH)_2_ and Zn(OH)_2_→ZnO + H_2_O or other byproducts (Bayaguud et al., 2022). In addition, while in alkaline electrolytes, the working mechanism of Zn anodes can be explained as Zn+4OH^−^ = Zn(OH)_4_^-^+2e^−^. The reaction chemistry of the parasitic HER can also be depicted as 2H_2_O+2e^−^→H_2_↑+2OH^−^, which occurred on the Zn anode surface accompanied by many other complex parasitic reactions (Tang et al., 2019).

In recent research of Zn-based batteries, RZBs in mild electrolytes are the main steam. The Zn-based batteries in an alkaline electrolyte that has not been fully investigated are briefly introduced in this review for future reference. Moreover, the HER and dendrite formation are both susceptible to the interface between the electrolyte and anode, as well as the surface condition of the Zn anode. The hydrogen evolution normally occurs from chemical/electrochemical reactions and causes the increased internal pressure of the sealed electric cells, leading to the failure of the sealing.

## Dendrite Inhibition Strategies

**FIGURE 1:**
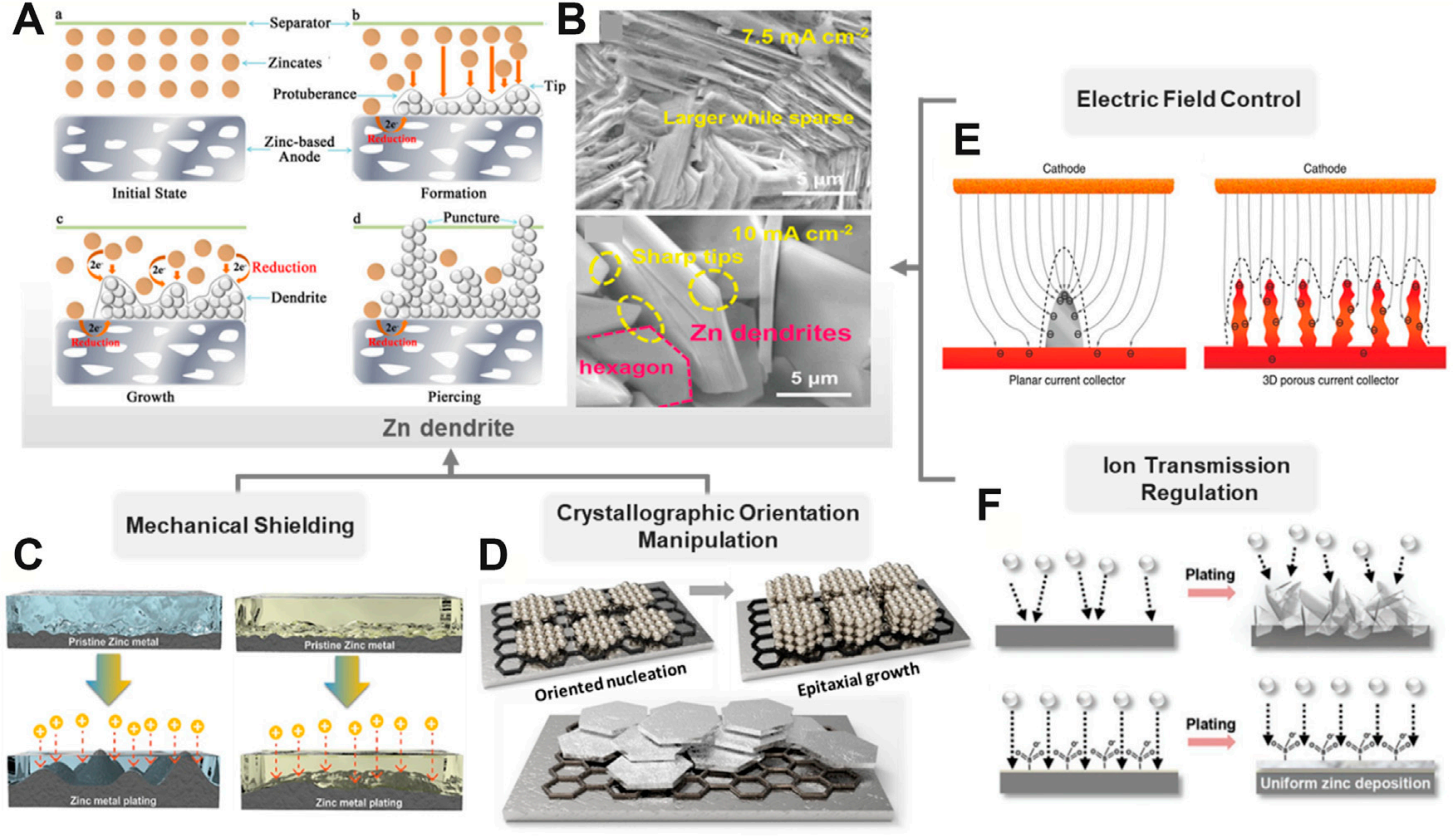
Schematic diagram of strategies for Zn dendrite suppression. **(A)** Fundamentals of Zn dendrite formation. (Lu et al., 2018) with permission from Wiley-VCH. **(B)** Scanning electron microscopy (SEM) images of the Zn dendrite. (Yang et al., 2019) with permission from Wiley-VCH. **(C)** Comparison of the morphology evolution of Zn foils with the liquid electrolyte and self-healable hydrogel electrolyte. (Ling et al., 2021) with permission from ELSEVIER. **(D)** Schematic illustration of the epitaxial metal electrodeposition of the Zn crystal. **(E)** Working mechanism and effect of the 3D current collector. (Yang et al., 2015) with permission from Springer. **(F)** Comparison of Zn deposition behavior while using the liquid electrolyte and a zwitterionic hydrogel electrolyte. (Mo et al., 2020) with permission from Wiley-VCH.

Exploring the suppression strategies of the Zn dendrite for uniform Zn deposition is beneficial to the cyclic stability and high Coulombic efficiency of rechargeable Zn-based batteries. Figure 1A illustrated the formation process of the Zn dendrite (Lu et al., 2018), and the surface micromorphology of deposited Zn reveals a flake distribution (Figure 1B) (Yang et al., 2019). During the charging–discharging cycles, the formation of the Zn dendrite is dominated by the mass transfer process mainly affected by the concentration gradient and the electric field distribution (Li et al., 2018). In addition, the dominant crystallographic orientation and the initial surface texture of the Zn anode also have great influence on the electrochemical performance, especially the rate and cycle capabilities. With the efforts of all parts concerned, a series of strategies have been proposed and categorized into four types: mechanical shielding, crystallographic orientation manipulation, electric field control, and ion transmission regulation.

### Mechanical Shielding

The Zn dendrites normally grow perpendicularly to the substrate and lead to the trend of a short circuit process. A shielding layer with high Young’s modulus will contribute to alleviating the dendrite growth from vertical evolution and lead to a better uniform deposition surface (Figure 1C) (Ling et al., 2021). From the mechanical perspective, physically mechanical shielding undoubtedly influences the dendrite growth by improving the interface strength to prevent battery failure. For instance, the solid-state electrolyte (SSE) can not only provide mechanical shielding with high strength but also avoid many parasitic reactions which often occur in aqueous counterparts (Wu et al., 2020). In general, SSEs include inorganic solid electrolytes (ISEs) and solid polymer electrolytes (SPEs). Research practices in the field of RZBs usually focus on the SPEs with moderate strength for the mechanical shielding layer. For instance, poly(ethylene oxide) (PEO) has attracted wide attention due to its good solubility to salts and relatively high ionic conductivity (Ma et al., 2020). In addition, the performance of ionic conductivity and mechanical stability of SPEs can be optimized by a combination of the PEO skeleton and functional fillers such as TiO_2_ nanoparticles, in which Zn^2+^ ions are transferred by the segmental motion of PEO chains, and the TiO_2_ nanoparticles contribute to the mechanical strength (Hiralal et al., 2010).

For RZBs, higher mechanical strength will further stabilize the Zn anodes theoretically. However, there are still no deep research and systematic simulation to quantify the threshold in the previous SSE research. Moreover, the poor interfacial contact and low ionic conductivity accompanied by high polarization are also becoming the burning issues. The physical shielding method is generally executed in coordination with other strategies in the strengthened solid-state electrolyte, separator, surface coating, and so on, and the most vital problem in developing strong mechanical shielding is how to balance the mechanical properties and electrochemical properties such as ion conductivity.

### Crystallographic Orientation Manipulation

At the beginning of electrodeposition, the deposited Zn nucleates randomly along various crystallographic orientations. The Zn crystals are subsequently formed and grow through the preferred crystallographic (001) facet due to their hexagonally close-packed orientated texture, resulting in hexagonal crystal plates in diverse directions (Han et al., 2020). Typically, the crystals which grow vertically to the substrate will form large dendrites and eventually pierce the separator, leading to battery failure. If the Zn crystal plates grow along the (002) direction, which are parallel to the substrate during electrodeposition, the resultant Zn anode with a dense and parallel-orientated texture would deliver better electrochemical/chemical stability (Zheng et al., 2019).

A lattice-fitting substrate for Zn deposition is critical for crystallographic orientations. During the epitaxial electrodeposition process, if the coherent or semicoherent interface layer between the deposited Zn layers and the substrate is formed and tends to grow parallelly to the substrate, a maximum interfacial compatibility and a minimal interfacial energy between the Zn-deposited layer and the substrate will be realized (Figure 1D) (Du et al., 2020). For instance, Archer et al. employed graphene, which possessed a low lattice mismatch with Zn, as an epitaxial deposition substrate to lock the crystallographic orientation of Zn plating (Zheng et al., 2019). The minimal lattice strain of the epilayer growth could facilitate the formation of a compact and flat interface for uniform Zn deposition.

The composition of the electrolyte that involves the salts, solvents, and additives is another important factor for the crystallographic orientations of Zn deposition. For example, sodium dodecyl sulfate (SDS) as the organic additive could effectively facilitate the Zn crystal growth along the (002) plane, thus resulting in a dense and orientated electroplating texture and thus extending the battery lifespan to over 1,000 cycles (Sun et al., 2017).

Apart from the substrate and electrolyte, the composition of a separator also influences the Zn crystal growth in a uniform manner. For example, a composite Nafion membrane was investigated and reported as an effective separator to regulate the Zn deposition behavior (Yuan et al., 2019). The abundant sulfate groups on the Nafion-based membrane could reshape the coordination of Zn^2+^ in an aqueous medium and contribute to the formation of a solid electrolyte interface (SEI) that is parallel to the substrate surface. This SEI was beneficial to the epitaxial Zn deposition and the oriented crystal growth, leading to a prolonged battery lifespan.

### Electric Field Control

Electric field is the driving force of ion transmission for initiating electrochemical reactions. With the elaborate structural design of electrodes or current collectors, the local areal current density will be decreased, leading to decreased polarization and uniform electric field distribution. This will alter the dendrite growth into a lateral trend, instead of vertical accumulation (Zou et al., 2018). For a conventional planar current collector, abundant small protrusions will be formed on the planar structure under the electric field due to 2D ion diffusion and nucleation and act as the favorable stripping points amplifying the subsequent deposition. When employing a 3D current collector such as the porous foam (Chamoun et al., 2015), sponge (Parker et al., 2017), micro-nanosheet (Zhao T. et al., 2018), and multi-channel metal lattices (Zhang et al., 2021), it was found that a uniform electric field could be initiated by the enlarged electroactive surface. The ion would nucleate and deposit in a homogenous way and gradually fill in the micropores within the 3D current collector, eventually eliminating the dendrite issues (Figure 1E) (Yang et al., 2015). Lu et al. developed a designed 3D Zn@CNTs with good affinity between the Zn layer and substrate, which displayed a stable stripping/plating cycling with over 200 h in a symmetric cell due to the homogenous precipitation of Zn (Zeng Y. et al., 2019). However, compared with the planar structure, the side reaction issues of the 3D structures are more severe due to their larger electroactive sites.

Employing charge/discharge protocols is an effective and cost-saving strategy to influence the dendrite growth by introducing preset *in situ* charging/discharging steps. Currently, an electrohealing strategy was developed to eliminate the existing dendrite by applying a cyclic stripping/plating process under the relatively low current density (Yang et al., 2019). Through this treatment, the concentration polarization surrounding the dendrite tips formed during current density cycling will be eased, and the sharp tips will, thus, be removed during stripping, resulting in a flat dendritic morphology and smooth surface. This strategy enabled the Zn symmetric cell a 516% prolonged lifespan even at 10 mA cm^−2^. This strategy is accessible without any special treatment, which provides a new perspective for battery maintenance.

### Ion Transmission Regulation

The ion transmission makes a significant difference in its nucleation barrier. In general, through regulating electrolyte components and concentration, the faster and more uniform ion migration can display a smaller nucleation polarization, which is conducive to the dendrite suppression (Zhang et al., 2016). Interfacial modification can stabilize the anode by alleviating parasitic reactions and providing an ion filter for uniform deposition in the meantime. Some functionalized separators will also regulate the ion flux motion for facilitating homogenous plating (Liang et al., 2020). Therefore, the strategies for ion transmission regulation can be developed from the following aspects: electrolytes, surface coatings, and separators.

The strategy of electrolyte optimization includes ion transfer facilitation and ion flux regulation. Normally, the salts with large anions in an electrolyte, such as Zn(CF_3_SO_3_)_2_ and zinc bis(trifluoromethanesulfonyl)imide (Zn(TFSI)_2_), contribute to a smaller potential hysteresis during Zn^2+^ plating/stripping than ZnSO_4_ or ZnCl_2_. This is because the large anions will attenuate the solvated sheath around Zn^2+^, leading to the lower ion-flux resistance (Sun et al., 2017). Hydrogel electrolyte, which possesses the homogenous polymeric matrix, can guide a uniform Zn ion flux and deposition (Figure 1F) (Mo et al., 2020). Similarly, some organic electrolyte additives such as polyethylene glycol (PEG) can be adsorbed on the anode surface and restrict the 2D Zn^2+^ diffusion, leading to uniform and dense Zn deposition (Mitha et al., 2018). Some surfactants such as sodium dodecyl sulfate and cetyltrimethylammonium bromide are also conducive to the ion transmission *via* selective adsorption, thus affecting the preferential orientation of Zn dendrites (Zeng X. et al., 2019; Liu et al., 2019).

Surface coating is a direct way to stabilize anodes prepared by spin coating, doctor blading, and atomic layer deposition, which can guide the Zn ion flux and facilitate a uniform Zn nucleation, thus realizing a fat Zn deposition layer (Li et al., 2020b; Deng et al., 2021). An ideal coating layer to prevent the dendrite growth should possess high ionic conductivity, long-term stability, and good compatibility with electrolytes. Normally, it can be clarified into inorganic and organic coatings. So far, CaCO_3_- (Kang et al., 2018), TiO_2_- (Zhao K. et al., 2018), ZnO- (Xie X. et al., 2020), and hafnium oxide (HfO_2_) (Li B. et al., 2021)-based inorganic coatings have been investigated and developed to guide the ion flux for uniform deposition. Nevertheless, their large weight will lower the power density (Li Q. et al., 2020). Organic coatings, such as PAM, polyvinyl butyral (PVB), 502 glue, and polyacrylonitrile (PAN), have been widely investigated and utilized as artificial SEIs for Zn anode stabilization, which exhibit abundant polar groups that can interact with solvated metallic ions (Li et al., 2022). These polymeric coatings could not only provide homogenous ion pathways and restrict the 2D diffusion of Zn^2+^ on the surface but also function like a shelter to block the detrimental side reactions. However, the mechanical stability of these polymeric coatings for dendrite suppression needs improvement (Rui et al., 2020).

The intrinsic properties of a separator such as elastic modulus, composition, and void uniformity will significantly influence the ion flux, resistance of ion transmission, and ion distribution. Through comparative study of the most commonly used separators, such as the filter paper, glass fiber, and polypropylene membrane, researchers found that these materials were difficult to achieve high elastic modulus, uniform ion distribution, low ion flux resistance, and good wettability at the same time (Xie C. et al., 2020). Hence, developing advanced separators is essential to prevent the dendrite growth and improve battery performance. Ghosh et al. reported a Zn^2+^-integrated Nafion-based ionomer membrane with superior mechanical strength and wettability, which can effectively guide the uniform ion distribution on the surface of the electrode (Ghosh et al., 2019).

## Hydrogen Evolution Reaction Suppression Strategies

**FIGURE 2:**
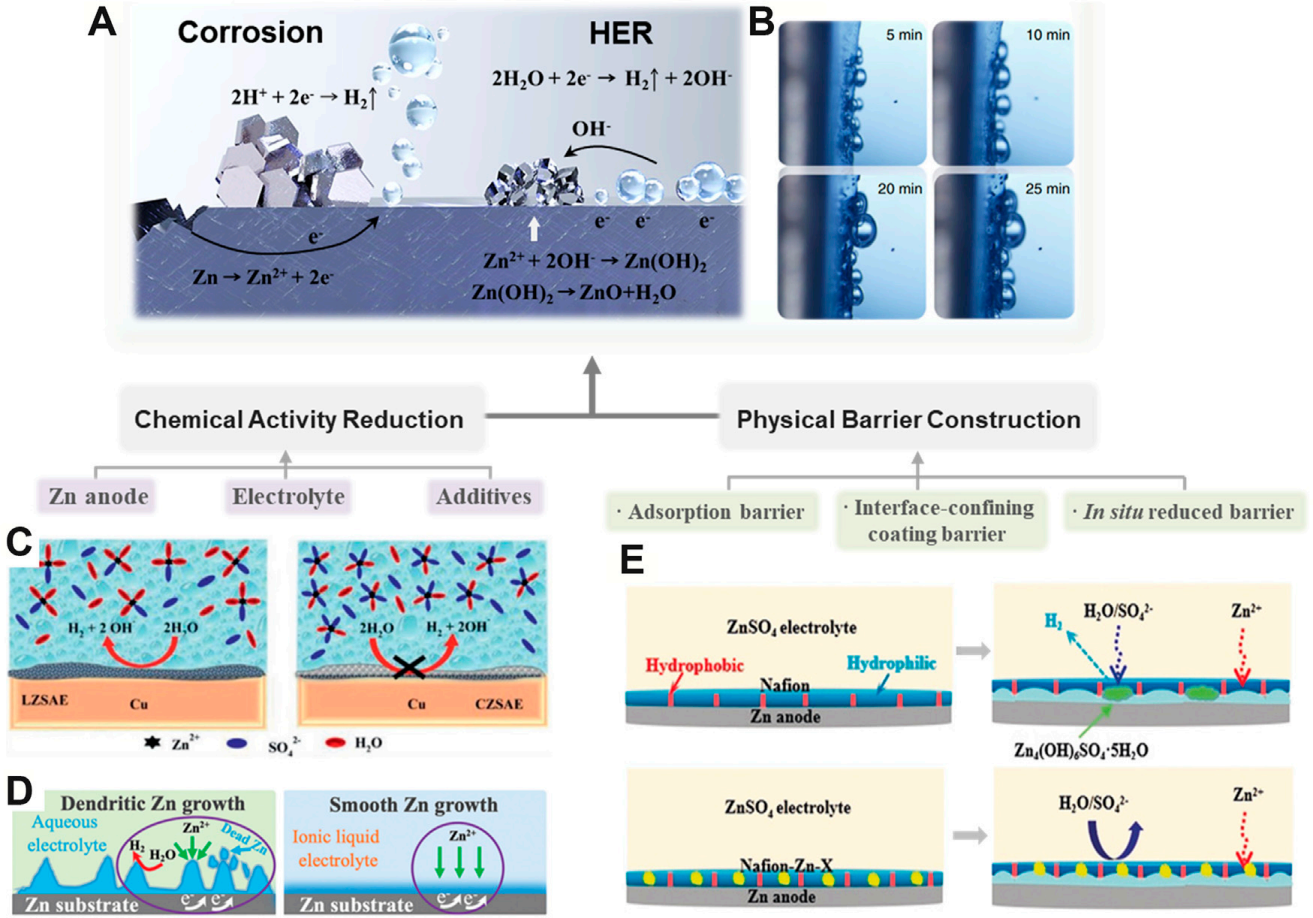
Schematic diagram of strategies for HER suppression. **(A)** Fundamentals of the HER and corrosion. **(B)** *In situ* optical micrograph of the HER. (Qiu et al., 2019) with permission from Springer. **(C)** High concentrated ZnSO_4_ electrolyte for HER suppression. (Olbasa et al., 2020) with permission from American Chemical Society. **(D)** Comparison of the HER and corrosion while using the aqueous electrolyte and ionic liquid electrolyte. (Ma et al., 2020) with permission from Wiley-VCH. **(E)** HER and corrosion inhibition effect of polyamide composite coating in the ZnSO_4_ electrolyte. (Cui et al., 2020) with permission from Wiley-VCH.

The HER is mainly related with the chemical activity of Zn metal and water, which is harmful to the battery performance of RZBs by causing corrosion and cell inflation (Figure 2A) (Bayaguud et al., 2022). Excessive hydrogen evolution may cause capacity deterioration and electrolyte leakage (Zhao J. et al., 2019). Substantial evidences have indicated that the HER can normally be mitigated from the thermodynamic and dynamic points such as manipulating the chemical activity and constructing a physical barrier (Yi et al., 2021).

### Chemical Activity Reduction

The activity of the Zn anode plays a vital role in the appearance of spontaneous corrosion and the HER catalytic activity during Zn stripping/plating (Figure 2B) (Qiu et al., 2019). To this end, optimizing the composition of the Zn anode can increase the corrosion resistance by enlarging the overpotential threshold of the HER (Shivkumar et al., 1998). In Zn–air batteries, some heavy metals such as Pb, Hg, and their oxides have been widely utilized as additives to suppress Zn corrosion, but the heavy metal pollution issues they brought cannot be ignored (Lee et al., 2006; Huang & Yang, 2015). Hence, some eco-friendly metallic compounds containing Al, Ni, In, and their oxides/hydroxides have been developed. For example, a Zn–Al–In hydrotalcite-like metallic compound which possesses a high overpotential of the HER was fabricated as a Zn anode (Wang et al., 2014). This composite anode enables the overpotential of the HER shift positively, thus inhibiting the corrosion of the anode. In addition, a series of metal oxides (e.g., Al_2_O_3_, Bi_2_O_3_, In_2_O_3_, etc.) have been introduced into Zn anodes as additives to suppress the HER and corrosion issues (Lee et al., 2013). Considering the Zn/Al_2_O_3_ composites as an example, it was found that the Zn/Al_2_O_3_ anode exhibited the lowest HER rate, whereas the pure Zn anode displayed the highest one. This is ascribed to the higher HER overpotential induced by the incorporation of Al_2_O_3_.

Apart from modifying the Zn anode, engineering the electrolyte could also significantly influence the HER process. Typically, tuning the concentration of salts, solvent, and the additives in the electrolyte is a fundamental approach to regulate the kinetics and thermodynamics of the HER. Increasing the salt concentration can not only decrease the equilibrium potential of H_2_O/H_2_ but also alter the coordination of Zn^2+^ ions and reshape the solvation sheath, which are favorable for HER suppression (Figure 2C) (Olbasa et al., 2020). For instance, the concentrated ZnCl_2_ with 30 mol L^−1^ could facilitate the coordination between Zn^2+^ ions and water molecules to form [Zn(OH_2_)Cl_4_]^2−^ and [ZnCl_4_]^2−^ instead of the [Zn(OH_2_)_6_]^2+^, thus ameliorating the HER and anode corrosion (Zhang et al., 2018). Similarly, a “water-in-salt” electrolyte containing 1 M Zn(CF_3_SO_3_)_2_ and 21 M LiN(CF_3_SO_2_)_2_ was reported. This electrolyte could enable the corrosion potential to shift positively from −0.974 to −0.768 V based on Tafel plots, which was due to the constrained activity of water. As a result, the corrosion and HER are restrained (Wan et al., 2019). In addition, several organic solvents such as acetonitrile and ethyl methyl carbonate have been widely used to dissolve a variety of Zn salts (e.g., Zn(ClO_4_)_2_, Zn(TFSI)_2_, and zinc trifluoromethanesulfonate (Zn(TfO)_2_).) for preparing potential non-aqueous electrolytes, which displayed a wide electrochemical stability window in RZBs (Wang et al., 2020; Pan et al., 2018). Ionic liquid is another important category of non-aqueous electrolytes to eliminate the HER, which features high-temperature stability and good compatibility with Zn salts. Recently, the 1-ethyl-3-methylimidazolium tetrafluoroborate (ILZE) electrolyte dissolved with 2 mol L^−1^ Zn(BF_4_)_2_ was developed, which showed excellent HER and Zn dendrite suppression properties during prolonged cycling (Figure 2D) (Ma et al., 2020).

Adding functional additives into an aqueous electrolyte can also effectively stabilize the H_2_O molecules for HER suppression. Some inorganic oxides (e.g., fumed) such as silica have been employed as an electrolyte additive to suppress the HER because they can form strong interaction with water molecules through hydrogen bonds (Huang et al., 2019). Moreover, some organic additives such as polyacrylamide (PAM) could decrease the Zn nucleation barrier, thereby leading to a compact and dense Zn deposition, which is beneficial to the HER and corrosion suppression (Zhang et al., 2019).

Increasing the salt concentration not only can suppress the HER but also can increase the ionic conductivity in a certain content. However, high salt concentration may bring some undesirable parasitic reactions, an increased cost, and the salting out effect. In addition, by utilizing the organic solvents, the property of HER inhibition will be very effective due to its water-free characteristic. However, the ionic conductivity of most organic solvents is always unsatisfactory, and the safety issues such as flammability and toxicity still need to be solved. For the additive employment, the ionic conductivity of the electrolyte and cost would not be seriously affected due to the low content of additives in the electrolyte, but the long-term stability of HER inhibition of utilizing additives may not be as good as the aforementioned two strategies.

### Physical Barrier Construction

Constructing a physical barrier between the electrode and electrolyte is a direct and effective way to ameliorate the HER and corrosion in an aqueous electrolyte through physically isolating the H_2_O molecules from the anode. Based on the working mechanisms, the reported barriers can be divided into three categories as discussed in the following section.

The first type is the adsorption barrier, which is operated by the interaction between the functional groups in the barrier and substrate surface. For instance, a small amount of 10 mmol of tricine(N-(tri(hydroxymethyl)methyl)glycine) was employed as the corrosion inhibitor to protect the Zn anode, which showed over 90% inhibition efficiency (Nady, 2017). This was ascribed to the fact that the inhibitor molecules physically interacted on the Zn anode could alleviate the formation of the corrosion product of Zn_5_(OH)_8_Cl_2_·H_2_O. In addition, some organic molecules and surfactants can also inhibit the HER and Zn corrosion through surface adsorption as discussed in the “Chemical Activity Reduction” section (Sun et al., 2017).

Similar to the SEI layer in a lithium ion battery, an *in situ* reduced barrier which contains anions with variable valence can be applied in RZBs to construct a protective layer for HER suppression. For example, the electrolytes containing ClO_4_^−^ could form an insoluble chloride layer onto the surface of the Zn anode due to its reduction during the repeated cycling process, which significantly prolonged the cycle lifespan of Zn anodes without any HER and corrosion by-products that could be detected by XPS characterization (Wang et al., 2019).

Furthermore, a suitable interface-confining coating barrier can also configure a chemically stable electrolyte/electrode interface to restrain the contact between free H_2_O molecules and the Zn anode surface, thus eliminating the parasitic reactions (Li C. et al., 2021; Shan et al., 2021). Recently, a MOF-based coating with abundant ion-transmission tunnels was developed for the Zn anode, which could spontaneously regulate the solvation structure to obtain a super-saturated electrolyte before Zn^2+^ reduction (Yang H. et al., 2020). The Zn^2+^·OSO_3_^2−^ ion could constrain water molecules after a partial desolvation process and subsequently transferred to the Zn anode through MOF channels with proper configuration, resulting in high electrochemical stability. In addition, some organic coatings such as polyamide (PA) (Zhao Z. et al., 2019), polymer glue (Jiao et al., 2021), and Nafion film (Cui et al., 2020), which normally show high adhesion to the substrate and low permeability of water molecules, can also serve as a HER barrier to protect the Zn anode from direct contact of water molecules, thus alleviating the undesirable gas evolution and Zn corrosion (Figure 2E).

## Conclusion and Outlook

In conclusion, although tremendous research has been made for developing high-performance RZBs, there are still persistent challenges needed to be conquered, especially the Zn anode issues involving dendrites and corrosion/HER issues. In this review, the fundamentals of the Zn dendrites and HER on Zn anodes have been briefly summarized accompanied with the associated reaction mechanisms. To address these issues, a series of strategies and the fundamental design principles for the dendrite and HER suppression are categorized and analyzed in-depth. To facilitate the deployment in practical applications of RZBs, the following suggestions and anticipations were proposed:

1. In general, the issues faced in practical applications are mutually exacerbated, and only employing a single optimization strategy cannot thoroughly eliminate all the problems. Hence, synergistic strategies with integrated functions combining the aforementioned methods to tackle multiple problems are highly desired. For instance, the economic hydrogel electrolyte can not only mediate the ion transmission but also serve as a thin surface coating to provide physical shielding for dendrite and corrosion prevention, thus improving the long-term stability.

2. Addressing one issue should not repose on deteriorating other properties. For example, applying 3D electrodes for dendrite suppression may expose a more specific surface for causing passivation and the HER, especially under harsh operating conditions.

3. The major advantages of RZBs compared to lithium batteries are cost-effectiveness, extremely intrinsic safety, and environment-friendly. Therefore, while exploring the organic electrolyte of RZBs for HER suppression, the aspects of toxicity and flammability should be carefully considered. Otherwise, the research for developing RZBs would be of limited significance.

4. While operating at a high temperature, the Zn corrosion and HER in RZBs would generally be aggravated due to its increased corrosion current and decreased HER overpotential. It is worth conducting the research regarding the adaptability and reliability of RZBs under extreme conditions.

5. In practical use, Zn corrosion and hydrogen evolution will also occur slowly in a standing stage on the Zn anodes. To reflect their practicability, apart from repeated charge–discharge test, the self-discharge test and storage test should also be performed on RZBs to evaluate the long-term stability of the aqueous RZBs precisely.
